# Psychological investigation in patients with polycystic ovary syndrome

**DOI:** 10.1186/1477-7525-11-141

**Published:** 2013-08-16

**Authors:** Fatemeh Bazarganipour, Saeide Ziaei, Ali Montazeri, Fatemeh Foroozanfard, Anoshirvan Kazemnejad, Soghrat Faghihzadeh

**Affiliations:** 1 Reproductive health and midwifery department, Tarbiat Modares University, Tehran, Iran; 2 Mental Health Research Group, Health Metrics Research Center, Iranian Institute for Health Sciences Research, ACECR, Tehran, Iran; 3 Gynecology and Obstetrics department, Kashan University of medical sciences, Kashan, Iran; 4 Biostatistics department, Tarbiat Modares University, Tehran, Iran; 5 Faculty of Medical Sciences, Zanjan University of Medical Sciences, Zanjan, Iran

**Keywords:** Anxiety, Depression, Quality of life, Polycystic ovary syndrome

## Abstract

**Background:**

The aim of the present study was to estimate the prevalence of mood disorders and examine a range of predictors for psychological well-being among Iranian women with polycystic ovary syndrome (PCOS).

**Methods:**

A cross-sectional study was undertaken to ascertain the factors related to psychological distress in PCOS patients in Kashan, Iran. Psychological distress was measured using the Hospital Anxiety and Depression Scale (HADS). In addition we assessed quality of life using the Short Form Health Survey (SF-36). Socio-demographic details and clinical information of PCOS including obesity (body mass index), excessive body hair (hirsutism score), acne, menstrual cycle disturbances, infertility and endocrine profile also were recorded for each patient.

**Results:**

In all 300 women with PCOS were entered into the study. Of these 32% (n =96) showed elevated HADS anxiety while depression was high in 5% (n =15). Quality of life was significantly impaired in women with anxiety (P <0.05), and depression (P <0.001) and in particular, in women with coexistence anxiety and depression (P <0.001) compared with unaffected participants. Compared with the non-depressed PCOS patients, the depressed women had significantly higher menstrual irregularities (P = 0.008). Moreover, we found significant difference in FAI level between the depressed and non depressed (p = 0.05), the anxious and non anxious patients (p < 0.001) compare to non affected PCOS women.

**Conclusions:**

The high prevalence of depression and anxiety in this population suggests that initial evaluation of all women with PCOS should also include assessment of mental health disorders. The clinician should further pay attention to background of their patients especially in view of the factors influencing psychological well-being.

## Background

Polycystic ovary syndrome (PCOS) is the most common endocrine disorder in women of reproductive age. It is estimated that 5 to 10% of women suffer from the condition [1]. The symptoms typically associated with PCOS, including amenorrhea, oligomenorrhea, hirsutism, obesity, infertility, anovulation and acne, can lead to symptoms of depression, marital and social maladjustment and impair sexual functioning [2].

The prevalence of depression in women with PCOS is high and varies from 28 to 64% [3-5]. The prevalence of anxiety in women with PCOS ranges from 34 [5] to 57% [6]. In particular, women with PCOS have been found to be at an increased risk of social phobia and suicide attempts [7]. The reasons for a higher prevalence of anxiety and depression in women with PCOS are likely to be complex. Some investigators suggest that physical symptoms experienced by women with PCOS are the likely cause of psychological distress [8,9]. However, evidence is inconsistent. While acne [6,10], hirsutism and BMI [2] have been linked to increased psychological distress in some studies, no link is demonstrated in others [11]. It is likely that multiple factors contribute to the high prevalence of both anxiety and depression in women with PCOS.

The aims of the present study were to estimate the prevalence of depressive and anxiety disorders among Iranian women with PCOS. We intended to examine a range of predictors for anxiety and depression including those relevant to the PCOS experience, such as BMI, infertility, androgens and also more general predictors, such as socio-demographic status. Further, we hypothesized that anxious or depressed PCOS women demonstrate reduced quality of life, particularly in the presence of coexistence disorders.

## Methods

### Design and data collection

This was a cross-sectional study of women with PCOS who attended two private gynecology clinics in Kashan, Iran from May to October 2012. Patients with confirmed diagnosis of PCOS were invited to participate in the study. After explaining the study objectives, written consent was obtained from each patient and they were requested to complete the study questionnaires. Patients were eligible if they met each of the following criteria: being 15–40 years of age; married; absence of non-classic adrenal hyperplasia, thyroid dysfunction, hyperprolactinemia, previous psychiatric diagnoses or using psychiatric medications including antidepressants; problems in speaking or listening Iranian; not taking any prescription medication (except allergy medications and occasional pain medications) for at least three months before entering the study; having two of the following Rotterdam diagnostic criteria: 1) Polycystic ovaries visualized on ultrasound scan (presence of 12 follicles or more in one or both ovaries and/or increased ovarian volume i.e., >10 ml), 2) clinical signs of hyperandrogenism (hirsutism score based on hirsutism score greater than 7 or obvious acne) and/or an elevated plasma testosterone (testosterone >2. 0 nmol/l), 3) having an interval between menstrual periods >35 days and/or amenorrhea, defined as the absence of vaginal bleeding for at least 6 months (i.e. 199 days) [12]. The research presented here is part of a larger study.

### Measures

Psychological distress: the Hospital Anxiety and Depression Scale (HADS) was used to assess depression and anxiety. The HADS is a self-administered instrument consisting of 14 questions. The instrument has two subscales including anxiety (seven items) and depression (seven items). All items are rated on a Likert scale ranging from 0 to 3. Sum scores <8 indicate normal range; scores from 8 to 10 reflect mild alterations and scores ≥11 indicate clinical relevance of symptoms [13]. A validation study of an Iranian version of the HADS indicated the translated version is reliable and valid measure of psychological symptoms in Iranian patients [14].

Quality of life: We used the Short Form Health Survey (SF-36) to examine quality of life in our study. The SF-36 is a well-known generic HRQOL measure and includes eight subscales: physical functioning (PF), role limitations due to physical problems (RP), bodily pain, general health perception (GH), social functioning (SF), role limitations due to emotional problems (RE), vitality (VT), and mental health (MH). The score for each subscale range from 0 to 100 where higher scores indicate better conditions. The Iranian version of SF-36 showed a good validity and reliability [15].

### Clinical symptoms of PCOS

1. Menstrual history: interval between menstruation during preceding 12 months were asked all patients and categorized to < 21 days, 21-34 days, 35-60 days(oligomenorrhea), > 199 days (amenorrhea) and variable.

2. Reproductive history: pregnancy history was asked categorized into the following: has been pregnant: all births, no losses; has been pregnant: some births, some losses; never pregnant; has been pregnant: no births, all losses.

3. BMI: weight and height were calculated by weight/ height squared [kg/m^2^] in all patients.

4. Body hair: Clinical assessment of hirsutism was determined using the Ferriman-Gallwey Scoring System (F/G score). Nine body sites (the upper lip, chin, chest, upper back, lower back, upper abdomen, lower abdomen, arm, and thigh) were graded from 0 (no terminal hair) to 4 (severe hirsutism). Scores can range from 0 to 36. A score of 7 or above was considered positive for hirsutism [16].

5. Acne: the Global Acne Grading System (GAGS) was used to determine acne. The GAGS considers six locations on the face and chest/upper back, with a factor for each location based toughly on surface area, distribution, and density of pilosebaceous units. The borders on the face are delineated by the hairline, jawline, and ears. The chest and upper back have been included because their involvement is critical in order to assess the severity of acne and to decide upon treatment. Each of the six locations is graded separately on 0-to-4 scale, with the most severe lesion within that location determining the local score. The global score is a summation of all local scores [17].

### Socio-demographic status

The study used years of formal education as a measure of socioeconomic status and it was categorized into five levels: no education, first level (1 to 5 years), second level (6–9 years), third level (10–12 years) and fourth level (more than 12 years). Studies from Iran showed that education could be a good proxy measure for socioeconomic status for Iranians [18].

### Laboratory measures

An overnight 8-hour fasting venous blood sample was obtained from each patient. Serum total testosterone (TT), sex hormone-binding globulin (SHBG), follicle-stimulating hormone (FSH), and luteinizing hormone (LH) were concomitantly assessed in all participants by ELISA (DRG Instruments GmbH, Marburg, Germany). TT and SHBG were used to calculate the FAI, which is presumed to be a useful indicator of abnormal androgen status [19]. FAI was estimated as TT (nmol/l)/SHBG (nmol/l) × 100. Except for amenorrhoeic women, all laboratory determinations were performed in the early follicular phase of the cycle. In amenorrhoeic women, after roll out of pregnancy the all laboratory determinations were performed.

### Data analysis

Data are presented as number and percentage unless otherwise indicated. Group comparisons were carried out with Student’s t-test, ANOVA and Chi square test where necessary. Post hoc Scheffe’ test was conducted for comparisons of individual means. P < 0.05 was accepted as significant. Statistical analysis was performed using Statistical Package for the Social Sciences 21.0 (SPSS Inc., Chicago, IL, USA).

### Ethics

The Ethics Committee of the Tarbiat Modares University approved the study. All patients give informed consent.

## Results

### Socio-demographic characteristics and clinical symptoms

In all, 300 women with PCOS were included in the study during the six months enrollment. The mean (SD) age of patients was 26.5 (4.44) years. The majority of women had education beyond high school (72.7%, n = 218). More than two-thirds of patients had never been pregnant nor had successfully carried a pregnancy to term and have abnormal menstruation. Socio-economic and clinical characteristic of the patients are presented in Table 1.

**Table 1:** Socio-demographical and (bio) clinical characteristics of PCOS patients

***Demographical***	
Age (years)^*^	26.56 ± 4.44
Education (years)^**^	
The first level (1 to 5 years)	32(10.7)
The second level (6–9 years)	50(16.7)
The third level (10–12 years)	126(42)
The fourth level (more than 12 years)	92(30.7)
Duration of marriage^*^	10.02 ± 4.20
***Clinical***	
Hirsutism score*	6.7 ± 5.73
Acne score*	10.54 ± 7.26
Interval between menstruation (days)**	
< 21	8(2.7)
21-34	109(36.3)
35-60	19(6.3)
>199	31(10.3)
Variable	133(44.3)
Reproductive history**	
Never pregnant	193(64.3)
Has been pregnant: all births, no losses	32(10.7)
Has been pregnant: some births, some losses	17(5.7)
Has been pregnant: no births, all losses	58(19.3)
BMI (kg/m2)**	
< 25	130(43.3)
25–30	120(40)
> 30	50(16.7)
***Endocrine***	
LH (IU/l)^*^	8.28 ± 6.16
FSH (IU/l)^*^	6.09 ± 4.42
Testosterone (nmol/L)^*^	1.24 ± 0.23
SHBG (nmol/L)^*^	55.57 ± 43.87
FAI^*^	10.21 ± 34.45

^*^Mean ± SD.

^**^N (percent).

### Prevalence of depression and anxiety and comparison for quality of life

**Figure 1:**
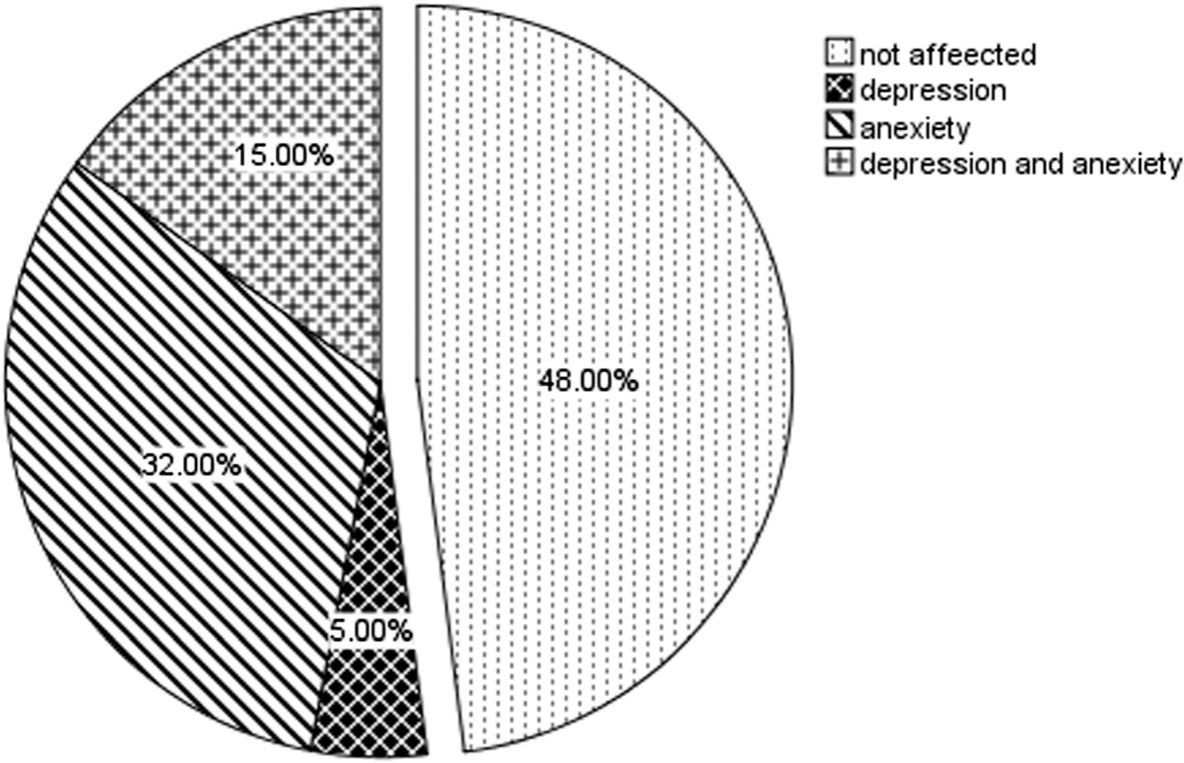
Frequency distribution of participants presenting with normal HADS (unaffected, anxiety, depression, or coexistence anxiety and depression.

Of the participants, 32% (n = 96) demonstrated elevated HADS anxiety scores (i.e. HADS anxiety subscale ≥11) and 5% (n = 15) showed elevated HADS depression scores (i.e. HADS depression subscale ≥11). There was 15% (n = 45) who scored above the cut-offs (≥11) for both anxiety and depression (Figure 1).

**Figure 2:**
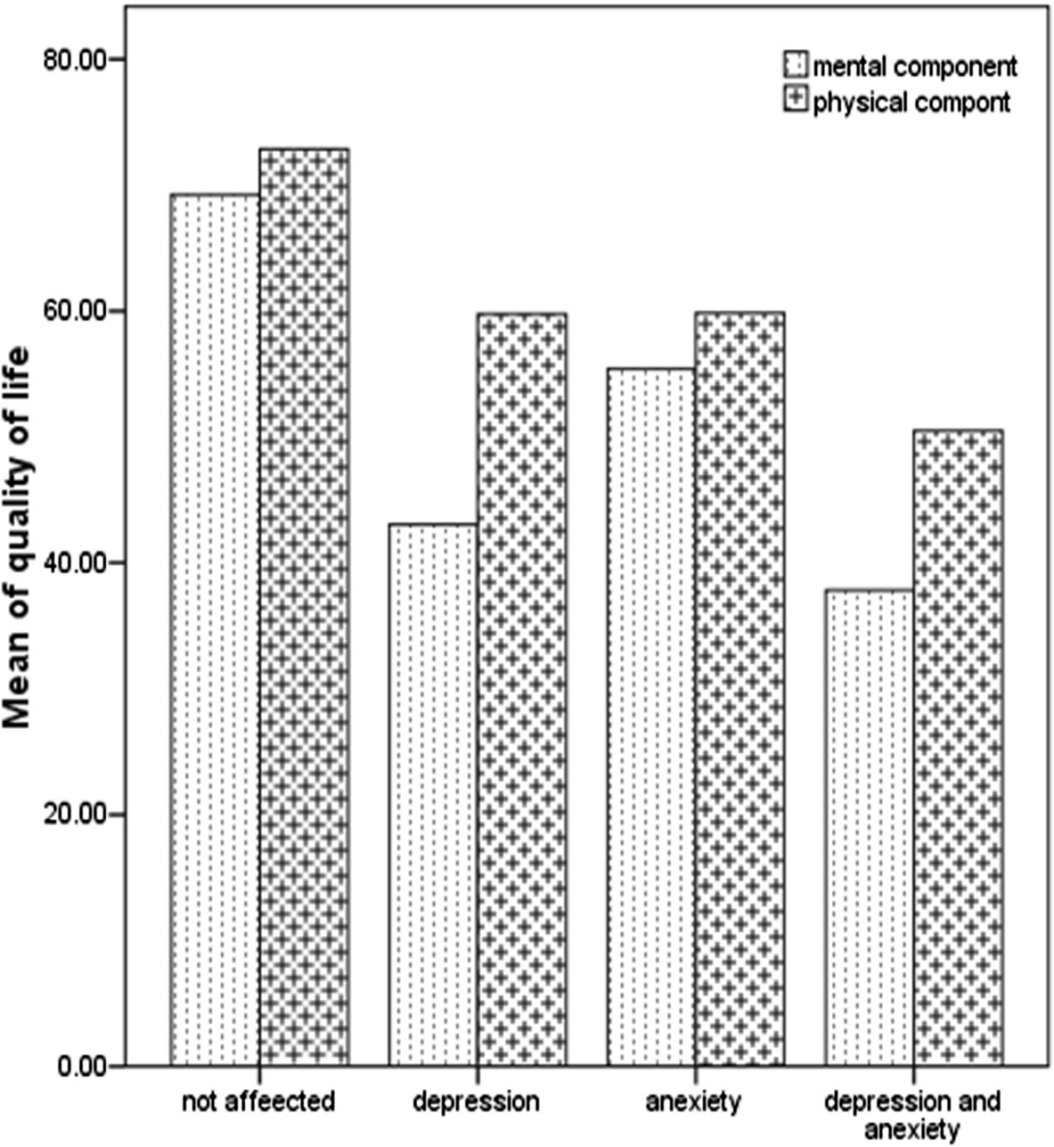
Comparison of sum scores of quality of life based on psychological function (unaffected, anxiety, depression or coexistence anxiety and depression) in PCOS patients.

Subsequently, to assess the impact of depression and anxiety symptoms on quality of life, the sample was divided into four subgroups based on the HADS scores (Figure 2). All groups with higher score on the HADS demonstrated markedly reduced psychological quality of life (F = 42.99, P < 0.001, Figure 2). Post hoc comparisons revealed a significantly lower quality of life in PCOS women with depression (P <0.001) and with anxiety (P < 0.001) compared with unaffected participants. The lowest mental health quality of life however, was observed in participants with coexistence anxiety and depression (post hoc Scheffe test for patients with anxiety and depression versus unaffected PCOS: P < 0.001).

Moreover, all groups with higher scores on the HADS demonstrated markedly reduced physical quality of life (F = 25.109, P < 0.001, Figure 2). Post hoc comparisons revealed a significantly lower quality of life in PCOS women with depression (P =0.04) and with anxiety (P < 0.001) compared with unaffected participants. The lowest physical quality of life, however, was observed in participants with coexistence anxiety and depression (post hoc Scheffe test for patients with anxiety and depression versus unaffected PCOS: P <0.001).

### Impact of socio-demographical and (bio) clinical characteristics of PCOS on anxiety and depression risk

To determine the association between socio-demographical and (bio) clinical characteristics of PCOS with depression and anxiety, the data was further analyzed based on presence or absence of depression or anxiety among patients. There was no significant difference in the mean age, BMI and other demographic characteristics within groups (Table 2).

**Table 2:** Comparison of psychological function base on socio-demographical and (bio) clinical characteristics of PCOS

		**Depression**		**Anxiety**	
		**Yes (n = 60)**	**No (n = 240)**	**Yes (n = 141)**	**No (n = 159)**
Age (year)^*^		26.71 ± 4.12	26.52 ± 4.52	26.85 ± 4.69	26.30 ± 4.20
*P value*		0.90		0.24	
Education (year)^*^		10.45 ± 3.57	11. 68 ± 3.38	10.98 ± 3.45	12.10 ± 3.39
*P value*		0.06		0.21	
Duration of marriage^*^		8.90 ± 4.33	10.29 ± 4.13	9.52 ± 4.01	10.46 ± 4.32
*P value*		0.91		0.41	
Menstrual irregularity^**^	Yes	43(22)	148(77)	91(47)	100(52)
	No	17(15)	92(84)	50(45)	59(54)
*P value*		**0.008**		0.76	
Infertility^**^	Yes	45(18)	193(81)	114(47)	124(52)
	No	15(24)	47(75)	27(43)	35(56)
*P value*		0.35		0.54	
BMI^*^		26.29 ± 3.87	26.28 ± 4.22	26.84 ± 4.23	25.80 ± 4.01
*P value*		0.28		0.63	
Hirsutism score*		6.86 ± 7.86	5.43 ± 5.16	6.26 ± 6.14	5.23 ± 5.48
*P value*		0.17		0.89	
Acne score*		9.20 ± 8.24	7.10 ± 7.60	8.41 ± 8.30	6.73 ± 7.19
*P value*		0.51		0.11	
FAI^*^		13.15 ± 46.99	9.47 ± 30.60	15.03 ± 47.28	5.94 ± 15.03
*P value*		**0.05**		**<0.001**	

^*^Mean ± SD.

^**^N (percent).

Compared with the non-depressed PCOS women, the depressed PCOS patients had significantly higher menstrual irregularities (P = 0.008). We found significant difference in FAI level between the depressed and non-depressed (p = 0.05)/anxious and non-anxious (p < 0.001) compared to non affected PCOS patients.

## Discussion

The goal of this study was to address the prevalence and determinants of psychological well-being in Iranian’s PCOS women. The main findings were the high prevalence of depression and anxiety in PCOS women and the reduction of quality of life in patients suffering from psychological impairment, especially in women who were suffering from both anxiety and depression. The prevalence of abnormal depression and mental health disorder varies among studies and this might be a reflection of the differences in methods and tools for screening and diagnosis, population differences, cultural difference and different classification systems. The prevalence of anxiety was high in these PCOS women and about one-third of the participants suffered from anxiety symptoms. This finding is consistent with recent study by Mansson et al. (2008) where they found a significantly elevated life-time incidence of social phobia in PCOS patients (27%; OR: 18.0, 95% CI = 2.2– 144), as well as a trend for an elevated incidence of generalized anxiety disorder (13%; OR: 7.3, 95% CI = 0.86–63) [7]. There is evidence to support the concept that anxiety is a risk factor for the development of depressive disorders [20,21]. About 5% of our participants showed elevated HADS depression levels, which is actually somewhat lower than the prevalence of depressive symptoms in previous studies reporting a range of 35 to 67% [22-24].

Our data indicated that a proportion of PCOS women (i.e. 15%) present with a coexistence of anxiety and depression. The risk of having coexistent depression and anxiety in women with PCOS is unknown. Only in one study found that 15% of PCOS patients showed having coexistent anxiety and depression [10]. Coexisting anxiety in depressed patients may worsen the outcome by increasing the risk of suicide, worsening overall symptoms, conferring a poorer response to treatment, increasing the number of medically unexplained symptoms, and increasing functional disability. Major depression is associated with patient suffering, disability, lost productivity and a higher mortality rate [25]. Untreated anxiety is also associated with high rates of medical co-morbidity and increased utilization of medical health care [26]. The majority of people with anxiety reported substantial interference with their life, a high degree of professional help seeking, and a high use of medication to relieve their symptoms. There is increasing evidence that depression may also be an antecedent to cardiovascular diseases [27] and depression predicts morbidity and mortality in patients with coronary artery disease (CAD) [28]. In other hand, Women with PCOS have several cardiometabolic risk factors increasing their overall risk for CVD.

In our study, participants with elevated HADS anxiety and depression levels reported significantly impaired psychological quality of life. This was especially true for those with coexistence anxiety and depression symptoms. It is difficult to know whether it is depression/ anxiety that influences lower perceived QOL or that poor perception of QOL increases depression/anxiety. Decreases in psychological, as well as in physical dimensions of quality of life in PCOS patients have previously been reported [29,30]. The determinants of reductions in quality of life in PCOS remain incompletely understood. However, there is converging evidence to suggest that physical aspects of quality of life may be best predicted by obesity and hirsutism, whereas psychological components of quality of life may be more closely related to psychological impairment [2,31,32]. The results confirm Adali΄s and Hirschberg´s findings [6,32,33], suggesting that treatment of PCOS should tackle both physical and psychological complaints. This is because psychological distress reduces motivation, and yet good motivation is the key to comply with medication and dietary management of PCOS [23].

Comparison between psychological well-being in women suffering from PCOS showed no significant difference in socio-demographic parameters; therefore, age, marital status, education had no influence on the prevalence of depression or anxiety in women with PCOS or their mental health status. Bhattacharya et al., also noted the same finding [3].

The current study established that women with menstrual irregularities seemed to be more depressed compared with women with normal menstruation. It has been shown that not only visual features of PCOS such as a higher body weight and an excessive growth of body hair were related to an increased experience of fear of what other people thought about their appearance, but also the absence of their cycle (amenorrhea) was negatively associated with fear of appearance evaluation [34]. The association between fear of negative appearance evaluation and non-visual characteristics might be explained by a reduced feeling of femininity [35]. The importance of menstrual irregularities for Iranian’s PCOS women has been demonstrated in previous study and have been found that menstrual problems were the greatest concern reported by the PCOS women [36]. Menstrual irregularities can have important social consequences, especially in many Muslim countries. For example, the tenets of Islam decree that menstruating women cannot pray [37]. Moreover, menstrual irregularities are strongly related with infertility. However, some socio-cultural generalizations are possible: the social pressure to have a child shortly after marriage is strong in the Iran.

The relationship between androgens and mood in women is controversial. Accordingly, we observed that patients with high level of FAI had an elevated risk for anxiety and depression. Conversely, Barry and colleagues [38] failed to find an association between testosterone and mood disturbances in women with PCOS. Accordingly, others also failed to find an association between depression and hormonal and metabolic profile [39,40]. Livadas et al. [39] studied whether anxiety was associated with hormonal and metabolic profile. PCOS women with higher anxiety scores showed significantly elevated HOMA-IR (insulin resistance) and FAI (free androgen excess) values than PCOS women with lower anxiety scores, independently of BMI. In the same line, the relation between greater FAI values and greater levels of anxiety was previously reported by Mansson et al. [7]. Perhaps studies with larger samples could elucidate this pattern much better.

It should be noted that this study have certain limitations. The study of patients with PCOS who were attending two private gynecology clinics may limit generalization of the findings to the entire PCOS population. Moreover, all of the patients in this study were married for cultural reasons (sex and infertility) in Iran. Therefore, the results of the present study have to be interpreted with some caution. Another limitation to this study is that the disease- specific scale available to measure the HRQOL of women with PCOS (MPCOSQ) was not used. The generic instrument used for the evaluation of the HRQOL in PCOS in the previous studies was generally the SF-36. However; we accept that this approach might not have been as sensitive for assessing the HRQOL scores when compared with the MPCOSQ. Therefore, the comparison with the MPCOSQ for Iranian PCOS women is necessary for future studies.

Generally, the contribution of individual PCOS symptoms to depression and anxiety risk reported herein should be interpreted with caution since most patients presented with a combination of PCOS symptoms, which may further enhanced the risk for anxiety. Clearly, since we did not analyze the effects of various combinations of clinical symptoms, this aspect of our analysis should be regarded as explorative. Possible selection bias, use of a screening tool alone without further diagnostic evaluation of depression, and lack of direct comparison with a healthy control group should be considered in interpretation of these results.

## Conclusion

PCOS is closely associated with psychological impairment with important implications that necessitate diagnosis and treatment of the disorders. The high prevalence rate of depression and anxiety in this population suggests that initial evaluation of all women with PCOS should also include assessment of mental health disorders. Psychological support assumes an important role in the management of the affected patients. This should not suggest that medical treatment of PCOS is not required but a thorough cooperation between medical treatment and psychological support would improve the situation PCOS affected women. Increased level of free androgen and history of menstrual irregularities were associated with mood dysfunction. The clinician should further pay attention to religious and cultural background of their patients especially in view of the factors influencing psychological well-being.

## Competing interests

The authors declare that they have no conflict of interest.

## Authors’ contributions

All authors were involved in designing of the study, data collection and analysis, interpretation of results and manuscript preparation. All authors read and approved the final manuscript.
